# Medication Use and Clinical Outcomes by the Dutch Institute for Clinical Auditing Medicines Program: Quantitative Analysis

**DOI:** 10.2196/33446

**Published:** 2022-06-23

**Authors:** Rawa Kamaran Ismail, Jesper van Breeschoten, Silvia van der Flier, Caspar van Loosen, Anna Maria Gerdina Pasmooij, Maaike van Dartel, Alfons van den Eertwegh, Anthonius de Boer, Michel Wouters, Doranne Hilarius

**Affiliations:** 1 Division of Pharmacoepidemiology and Clinical Pharmacology University of Utrecht Utrecht Netherlands; 2 Dutch Institute for Clinical Auditing Leiden Netherlands; 3 Stichting Volksgezondheidszorg (VGZ) Arnhem Netherlands; 4 Dutch Association of Hospital Pharmacists De Meern Netherlands; 5 Medicines Evaluation Board Utrecht Netherlands; 6 Department of Medical Oncology Amsterdam University Medical Center, location VUmc Amsterdam Netherlands; 7 Department of Biomedical Data Sciences Leiden University Medical Centre Leiden Netherlands; 8 Rode Kruis Ziekenhuis Beverwijk Netherlands

**Keywords:** real-world data, quality of care, medicines, cancer

## Abstract

**Background:**

The Dutch Institute for Clinical Auditing (DICA) Medicines Program was set up in September 2018 to evaluate expensive medicine use in daily practice in terms of real-world effectiveness using only existing data sources.

**Objective:**

The aim of this study is to describe the potential of the addition of declaration data to quality registries to provide participating centers with benchmark information about the use of medicines and outcomes among patients.

**Methods:**

A total of 3 national population-based registries were linked to clinical and financial data from the hospital pharmacy, the Dutch diagnosis treatment combinations information system including in-hospital activities, and survival data from health care insurers. The first results of the real-world data (RWD) linkage are presented using descriptive statistics to assess patient, tumor, and treatment characteristics. Time-to-next-treatment (TTNT) and overall survival (OS) were estimated using the Kaplan-Meier method.

**Results:**

A total of 21 Dutch hospitals participated in the DICA Medicines Program, which included 7412 patients with colorectal cancer, 1981 patients with metastasized colon cancer, 3860 patients with lung cancer, 1253 patients with metastasized breast cancer, and 7564 patients with rheumatic disease. The data were used for hospital benchmarking to gain insights into medication use in specific patient populations, treatment information, clinical outcomes, and costs. Detailed treatment information (duration and treatment steps) led to insights into differences between hospitals in daily clinical practices. Furthermore, exploratory analyses on clinical outcomes (TTNT and OS) were possible.

**Conclusions:**

The DICA Medicines Program shows that it is possible to gather and link RWD about medicines to 4 disease-specific population-based registries. Since these RWD became available with minimal registration burden and effort for hospitals, this method can be explored in other population-based registries to evaluate real-world efficacy.

## Introduction

Regulatory authorities approve the majority (76%) of new cancer drugs based on evidence provided by randomized controlled trials (RCTs) [1]. These RCTs have high internal validity and are widely considered the gold standard for establishing the efficacy of new drugs [2]. Many new cancer drugs have been recently approved based on very specific patient groups, surrogate outcomes, and lower patient numbers; these drugs are increasingly approved in accelerated tracks [3,4]. The selected patient groups and well-controlled setting of these RCTs has led to criticisms of their external validity [5]. In addition, recent research has shown that almost one-half of RCTs that applied for marketing authorization for new cancer drugs in Europe had a high risk of bias. This increased risk of bias was caused by their design, conducted analyses, and conduct deficits [1]. Further, due to the increase in newly approved cancer and rheumatic disease drugs, health care costs have increased. The total expenditures by hospitals on expensive medicines in the Netherlands reached €2.1 billion (US $2.2 billion) in 2019 [6].

Following market entry, new cancer drugs are prescribed to a broader group of patients with different characteristics. This leads to a gap in clinical outcomes evidence between RCTs and the real world [7,8]. During routine clinical practice, real-world data (RWD) are generated and registered in validated population-based cancer registries. Clinical quality registries are an important tool for quality assessment and improvement in hospitals, consequently leading to demonstrable improvements in patient outcomes [9]. Comparing the quality of care across hospitals results in insights into differences in outcomes, which can lead to improvements in care [9,10]. Furthermore, data from quality registries are used for outcomes research and to study practice variation between centers using quality indicators [11]. Besides clinical quality registries, detailed administrative and declaration data are available specifically on the use of (expensive) drug treatments. The combination of these data in clinical quality registries, hospital administrative data, and declaration data of drugs used in these indications could be valuable to bridge the efficacy-effectiveness gap.

Previous initiatives linked various databases on drugs to clinical data. This linkage made it feasible to study drug use, health resource use, costs, effectiveness, and the safety of medicines [12]. However, a gap remains for recently approved expensive cancer drugs.

To better understand the effectiveness of expensive cancer medicines in a real-world population, the Dutch Institute for Clinical Auditing (DICA) initiated the Medicines Program in 2018. The program aims to identify variation in use and clinical outcomes of expensive medicines, provide postmarketing authorization data, provide a tool for clinicians to benchmark their practice on the use of expensive medicines, and stimulate interactions between clinicians to share best practices. In this program existing data sources were used. This study aims to describe the potential for the addition of declaration data to quality registries to provide participating centers with benchmark information about the use of medicines and associated outcomes.

## Methods

### Ethics Approval

In compliance with Dutch regulations, the DICA quality registries were approved by the medical ethical committee of the Leiden University Medical Center and was not subject to the Medical Research Involving Human Subjects Act.

### Data Sources

Different existing data sources were used in the DICA Medicines Program; these data sources were linked. The first data sources were national population-based registries that are managed by the DICA. The DICA is a nonprofit organization that facilitates 23 population-based registries on different disciplines and diseases. These registries include information on clinical characteristics but contain limited data on the use of medicines. The DICA Medicines Program uses the Dutch Colorectal Audit (DCRA) [10], the Dutch Lung Cancer Audit [13], and the National Breast Cancer Organization Breast Cancer Audit (NBCA) [14]. These quality registries include information on patient, tumor, and treatment characteristics, and are used to compare hospitals on structure, processes, and clinical outcomes [15,16]. A previous study has shown that the data entered in the DICA registries are accurate and complete [17].

The second data source was financial and administrative data, including hospital pharmacies’ declarations of expensive medicines for health insurers. These expensive medicines are listed as expensive (>€1000 per patient per year, equivalent to >US $1058.39) by the Dutch Healthcare Authority [18]. This data source includes precise and valid information about the diagnosis, date of prescription, dose, and quantity of a prescribed drug. Administrative data from hospitals include declarations for the reimbursement of expensive medicines. Only expensive medicines that were relevant and related to the diagnosis were linked to the clinical data.

The third data source includes the Dutch diagnosis treatment combinations (DBC) information system, which contains information on in-hospital activities, such as computerized tomography (CT) scans, infusions, hospital admissions, day treatments, and radiology treatments. The DBC information system is used for the registration and reimbursement of hospital and medical specialist care. This system was introduced in the Netherlands to increase the transparency of care. Furthermore, DBC information systems were initiated to create a supply-led system, increase efficiency, and facilitate competition between health care providers [19]. Because the DCRA and NBCA quality registries only include patients undergoing surgical operations, patients with metastasized cancers who do not undergo surgical operations are missing. To include patients with metastasized colorectal and metastasized breast cancer, the DBC data were used and linked to the fourth data source.

The fourth data source was survival data from the national claims database (VEKTIS) from health insurers [20]. VEKTIS is the national insurance database, which contains administrative data from Dutch national health care insurers, covering approximately 17 million individuals. By adding this data source, we could assess overall survival (OS) from diagnosis and the start of systemic therapy. Data were retrospectively collected from patients treated from 2017 to 2020. Although the DICA Medicines Program was established in 2018, data from 2017 were available from the hospitals and were therefore linked.

### Data Linkage and Privacy

**Figure 1:**
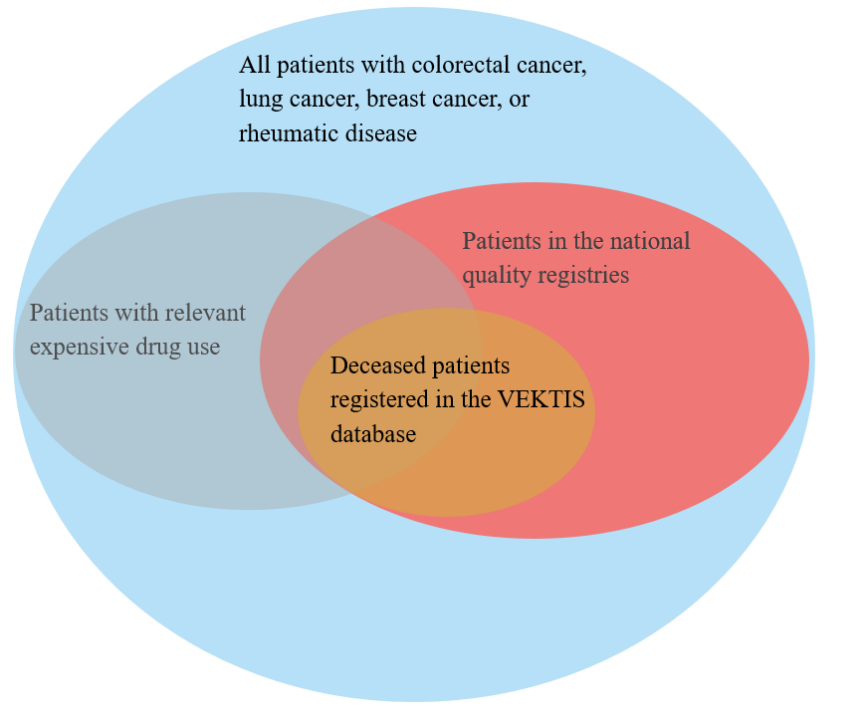
Visualization of the patients included in our study and the different data sources used.

The first step in data linkage was identifying patients diagnosed with colorectal cancer, lung cancer, breast cancer, and rheumatic disease using the DBC information system. The DBC information system is used for the registration and reimbursement of health care in the Netherlands. The second step was to identify whether these patients used relevant expensive drugs, and the third step was to determine whether these patients are registered in the national quality registry. Information on the date of death from the VEKTIS database was added for deceased patients (Figure 1). Data were linked based on hospital patients’ ID. A third party pseudonymized patient IDs. The results were visualized in dynamic web-based dashboards in which (systemic) treatments were linked to clinical parameters. Filters on patient and tumor characteristics, clinical outcomes, and therapy varied for the different diagnoses, depending on relevance. Furthermore, participating hospitals were compared, and practice variation was visualized and discussed to share knowledge on medical treatment differences.

### Statistical Analysis

The analyses in this manuscript are exploratory. Descriptive statistics were used to assess patient, tumor, and treatment characteristics. Time-to-next-treatment (TTNT) and OS were estimated with the Kaplan-Meier method. Survival times were calculated from the start of a systemic therapy until subsequent treatment (TTNT) or until death from any cause (OS). Patients who were alive or lost to follow-up were right censored at the time of their last registered expensive medicine use. All the statistical data were analyzed using R (version 4.0.2; R Foundation for Statistical Computing) within the RStudio environment (version 3.5.2; RStudio PB; packages tidyverse [21], TableOne [22], Survminer [23]).

## Results

### Database

**Figure 2:**
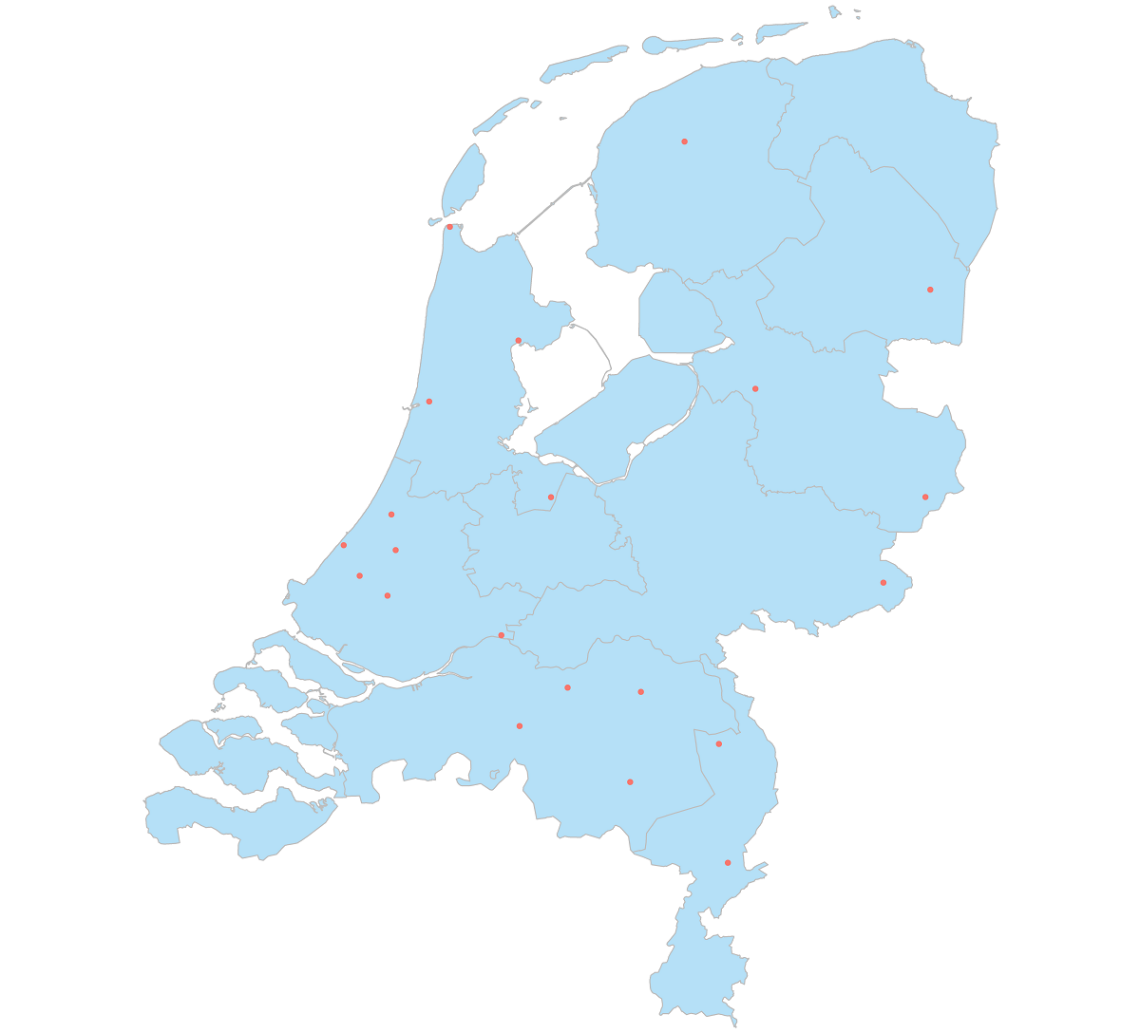
A map of the Netherlands including the geographic location of the participating hospitals in the Dutch Institute for Clinical Auditing Medicines Program (red dots).

A total of 21 Dutch hospitals participated in the DICA Medicines Program and were included in this study. Of these hospitals, 9 were top clinical hospitals, 11 were peripheral hospitals, and 1 was an academic hospital. The geographic location of these hospitals is shown in Figure 2, which indicates they are spread across the country. The DICA Medicines database included a total of 7412 patients with colorectal cancer, 1981 patients with metastasized colon cancer, 3860 patients with lung cancer, 1253 patients with metastasized breast cancer, and 7564 patients with rheumatic disease.

### Benchmarking

The DICA Medicines Program provides the ability to compare results between hospitals to improve the quality of care provided. Hospitals were provided with web-based dynamic dashboards (Multimedia Appendix 1), continuously comparing their data to the benchmark. The benchmark consisted of all other participating hospitals. An example of benchmarking is the use of systemic therapies at the end of life in patients with metastatic colorectal cancer. This varied between hospitals from 4.2% (5/119) to 27.8% (5/18), with a median of 13.4%. The dashboards also provide information on the type of systemic therapy used at the end of life. A signaling function is included in the dashboard if hospitals deviate from the benchmark (Multimedia Appendix 2). Deviation from the benchmark was defined as a ranked average calculated as follows: (Percentage of cases within hospital X – Percentage of cases within the benchmark)^2^ + Total number of patients in the benchmark.

### Use of Medicines and Patient Characteristics

The linkage of different data sources led to new insights into hospitals’ use of medicines and patient populations. The patient and tumor characteristics are listed for each medicine in the dashboard as a table that hospitals can compile with available variables. One of the participating hospitals discovered a deviation from the benchmark in the percentage of mesothelioma using the dashboard (Multimedia Appendix 3). This was 9.2% (14/153) for the specific hospital, compared to only 18% (105/3480) in the benchmark.

### Treatment Information

**Figure 3:**
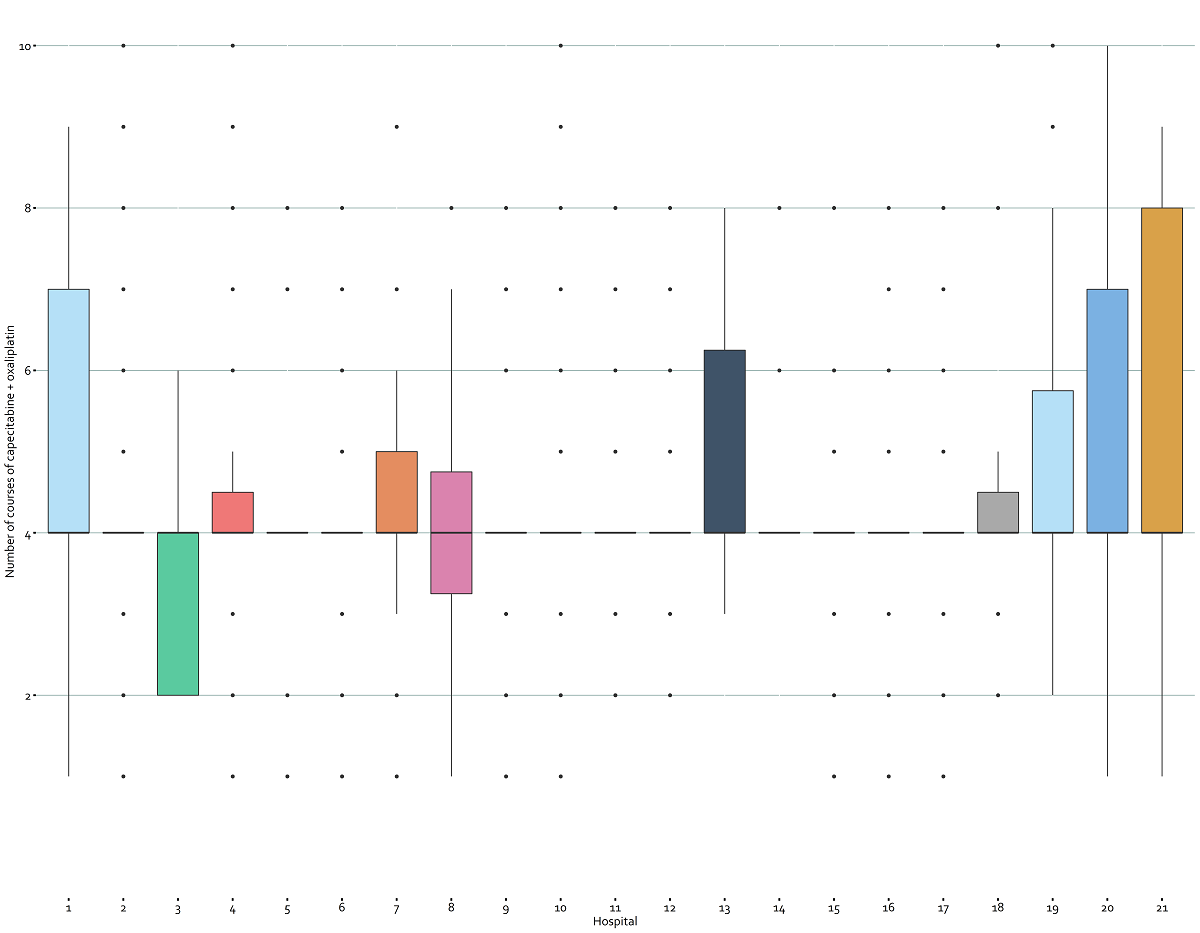
Number of courses of capecitabine + oxaliplatine for the adjuvant treatment of colorectal cancer patients per hospital.

**Figure 4:**
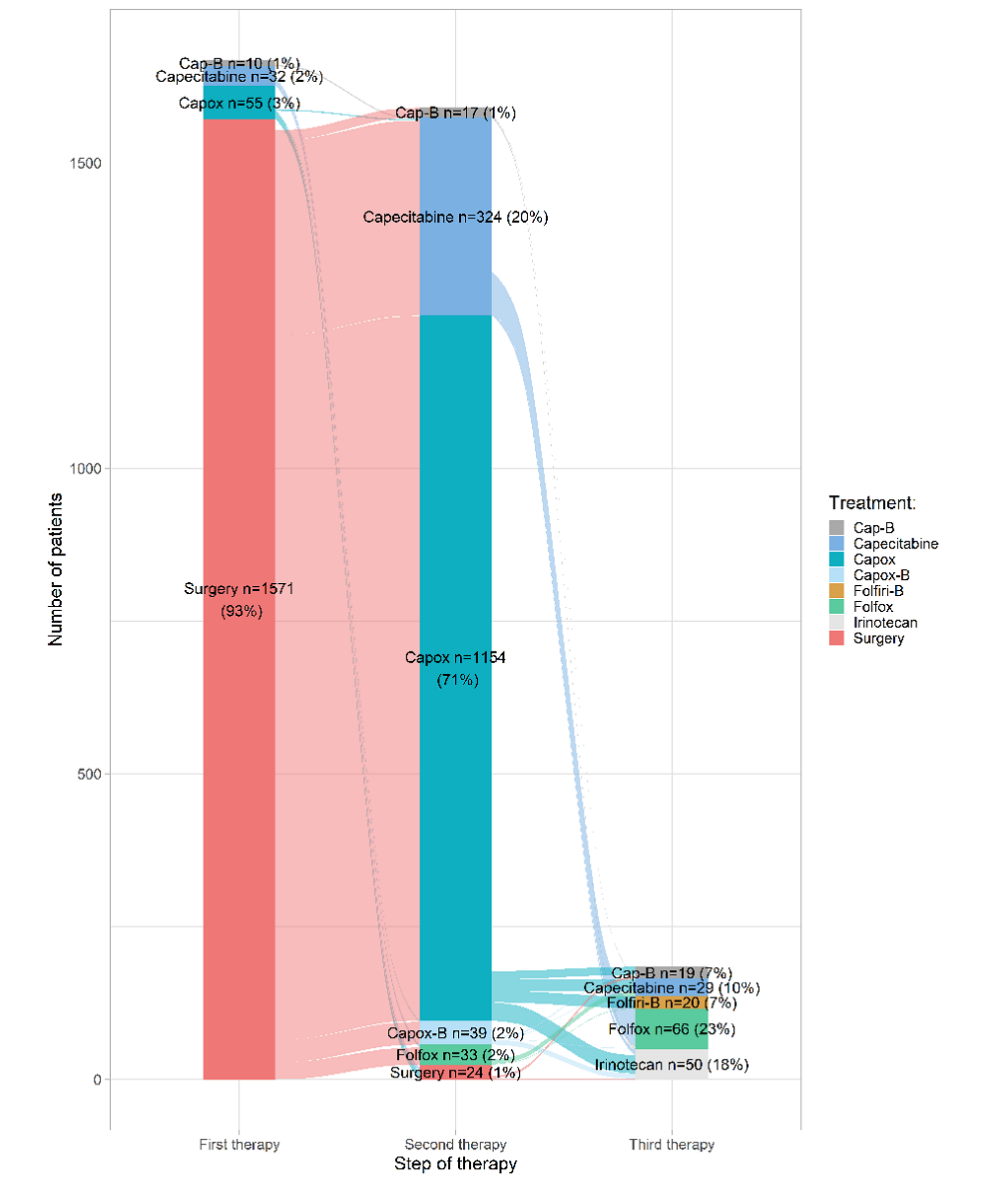
Treatment patterns in patients with stage III colon cancer treated between 2017 and 2020 (N=1668). The Sankey diagram shows the flow of patients from the first treatment step to the second treatment step and from the second treatment step to the third treatment step. The width of the lines corresponds with the number of patients. Systemic therapies with less than 5 patients are not displayed in this graph. Cap-B: Capecitabine plus bevacizumab; Capox: Capecitabine plus oxaliplatin; Capox-B: Capecitabine plus oxaliplatin plus bevacizumab; Folfiri-B: Fluorouracil plus irinotecan plus bevacizumab; Folfox: Fluorouracil plus oxaliplatin.

Linking clinical data to systemic treatment information also led to detailed information for each medicine, such as treatment duration in months and the number of cycles per patient. An example is the number of courses of capecitabine and oxaliplatin for the adjuvant treatment of colorectal cancer (Figure 3). Furthermore, administrative data were used to visualize treatment steps in Sankey diagrams in the dashboard, which can be adjusted for specific filters on patient, tumor, and treatment characteristics. Figure 4 shows the Sankey diagram for patients with metastasized colon cancer who were treated between 2017 and 2020. The dashboards also contain detailed information on diagnostic imaging (CT and magnetic resonance imaging scans), the number of consults (or teleconsults), clinical admissions, and emergency room visits pre- and post treatment for each medicine (Multimedia Appendix 4).

### Clinical Outcomes

**Figure 5:**
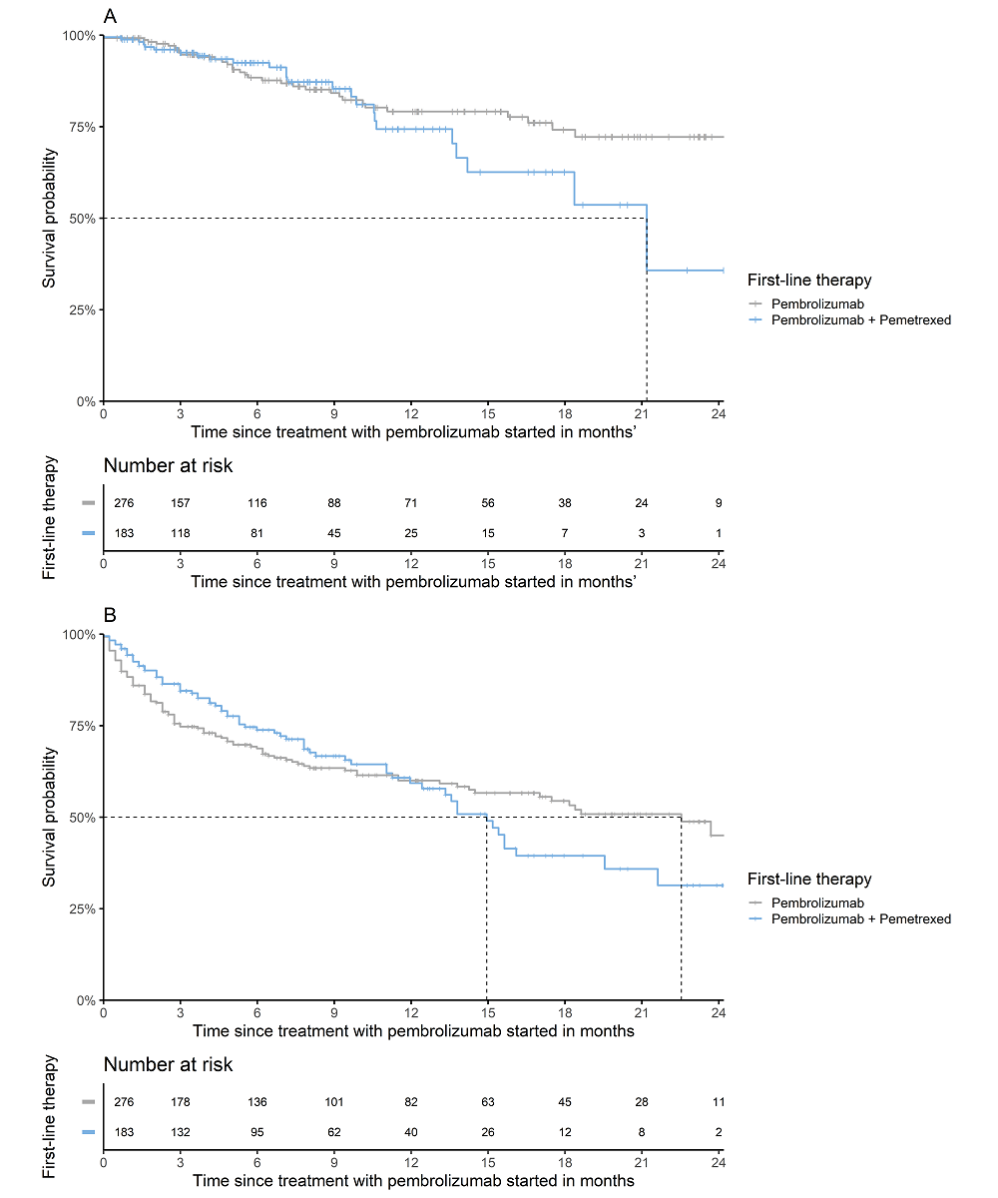
(A) Time-to-next-treatment of lung cancer patients treated with first-line pembrolizumab or pembrolizumab + pemetrexed between 2017 and 2020 and (B) overall survival of lung cancer patients treated with first-line pembrolizumab or pembrolizumab + pemetrexed between 2017 and 2020.

The DICA Medicines Program also provides hospitals with data on clinical outcomes, such as TTNT and OS. Figure 5 shows the TTNT of patients with metastasized lung cancer treated with first-line pembrolizumab or pembrolizumab and pemetrexed combination therapy. The median TTNT was 22.5 (95% CI 17.0, upper range not available) months and 14.9 (95% CI 12.4-21.6) months for pembrolizumab monotherapy and the combination of pembrolizumab and pemetrexed, respectively. The OS of these treatments is presented in Figure 5. Each hospital’s outcomes are compared to the benchmark. It is also possible to compare clinical outcomes between treatments in exploratory head-to-head comparisons for hospitals specifically or the benchmark in specific patient populations.

### Costs

Use of the financial and administrative database from hospital pharmacies provided us with access to detailed information on the costs of systemic therapies (total costs per treatment and costs per patient) in certain subgroups. Hospitals can upload their paid prices to the dashboard, which is then connected to the medicine and patient information (Multimedia Appendix 5). Prices paid by other hospitals are not shown due to confidential agreements between pharmaceutical companies and hospitals.

## Discussion

### Principal Findings

This paper reports on the initial results on the potential applications of data from the DICA Medicines Program; in this program, RWD are generated by linking 4 data sources, including data from quality registries, financial pharmacy data, in-hospital activities systems data, and reimbursement data from 21 Dutch hospitals. In this paper, we reported on the potential of this program in terms of benchmarking, treatment information, clinical outcomes, and costs. To be able to use the data as benchmark information, the data were visualized in web-based dashboards available to clinicians, insurers, and researchers; this led to insights on medication use, clinical outcomes, and costs without any additional registration burden for hospitals. Benchmarking hospital performance is relatively uncommon in the field of medical oncology in contrast to surgical oncology, where many quality registries exist that monitor the quality of care in every hospital [10]. Benchmarking information can support hospital pharmacists, oncologists, and other medical professions involved in the systemic treatment of patients to reach a certain level of care. RWD on the use and efficacy of systemic therapies are needed in daily clinical practice. As the real-world setting differs from the RCT setting, these data are needed after marketing authorization. This project provides real-world evidence, for which there is growing interest. One should be cautious when making definitive conclusions based on observational data. Minor observed differences could be the result of unknown confounding factors [24]. Other initiatives on the linkage of administrative data are similar and link patient-centered health data such as patient-reported outcome measures and clinical laboratory measurements but involve small patient groups [25] or limited patient and tumor characteristics [12].

### Strengths

First, data are validated at the time of delivery from the hospitals with the clinicians. A lot of effort is put into the validation of the algorithms that are used in the dashboards, for example, in building Sankey diagrams for treatment sequences in specific patient populations. The second strength of the DICA Medicines Program is the use of existing data sources, thereby minimizing the extra registration burden for medical specialists. This strategy could also be used by other parties to minimize registration burden and maximize the value of available RWD sources. Variables that could easily be derived from the declaration data were the number of expenses, start dates of medications, and the total dosages. Third, the program consists of many participating hospitals within a widespread geographic location, resulting in the inclusion of many patients, who are representative of the Dutch population. Another strength is the linkage of survival data to the other data sources. The database from the national health insurers is a valid source as health care insurance coverage stops when a patient dies. The final strength is that the data are up-to-date and representative of the current situation. This is especially valuable in situations such as the COVID-19 pandemic, where the systemic treatment of some patients with cancer was adjusted. Since the data are updated quarterly, it was possible to monitor the impact of COVID-19 in certain subgroups of patients in the dashboard. The DICA Medicines Program led to various insights into medication use. Questions related to the use of (expensive) medicines can be answered using the dashboards, in which users can select patient populations or treatments of interest.

### Limitations and Future Perspectives

In the clinical registries used for this study, some indications had incomplete data. The DCRA only includes patients undergoing surgical operations, which leads to incomplete clinical data on patients with metastasized cancers and colorectal cancers. This was also the case for patients with metastasized breast cancer. In this subpopulation of patients with breast cancer, essential tumor information, such as receptor status, is lacking. In addition, we are unable to extract information about weight, response status, date of progression, or toxicities from the declaration data. These are mostly data registered in unstructured text in electronical medical records. However, our intention is to complement the clinical data of these patient groups with other techniques that do not lead to further registration burden, such as text mining. Second, due to privacy regulations in the Netherlands [26], it is not permitted to follow-up on patients when they are referred to other hospitals for treatment. An individual patient may seek a second opinion from another hospital. This may have led to incomplete treatment information and individual patients being included twice in the database. Especially for university hospitals, where many patients are referred, it is necessary to have the complete treatment information. Previous analyses on the entire population of patients with lung cancer showed this was the case in <5% of all patients in the Netherlands. In this study, there may be an overestimate in the number of patients but not the number of prescriptions as these are validated declarations made by the hospitals. Third, more information on patient and tumor characteristics is needed to allow for head-to-head comparisons of medicines. Registries should therefore include information on response status and detailed treatment-related toxicity within each line of treatment. At this moment, emergency room visits and hospital admissions are linked to the use of medications and presented in the dashboards. However, these are only surrogate outcomes and do not give insight into the exact response or toxicity. Adding more outcomes of systemic therapies will also be an opportunity for surgical quality registries to become multidisciplinary, where both surgeons and medical oncologists register specific patients’ characteristics and outcomes. We are currently exploring text mining opportunities to add information on toxicities and response statuses to the quality registries.

Presently, hospitals use dashboards to benchmark their results against those of other hospitals and gain insights into the use of medications and patient populations, as we showed in this study. The dashboards can also be used for multiple other purposes and by different stakeholders in the future. First, dashboards and RWD can serve as communication tools between physicians and their patients. Based on specific patient and tumor characteristics, clinical outcomes can help patients better understand their disease course and improve shared decision-making. Second, registration authorities can also benefit from data as presented in this study. Data on newly approved medicines used in clinical practice are included in financial pharmacy data and can be linked to population-based registries. Especially for postapproval measurements, this information is valuable in monitoring the safety and effectiveness of medicines [27]. This can, in certain cases, eventually lead to the replacement of postapproval clinical studies, which will save time and financial resources. The European Medicines Agency and US Food and Drug Agency are increasingly interested in RWD for the evaluation of medicines [28,29]. Furthermore, health care insurers are interested in these data for reimbursement and effective use of expensive medications in the real-world setting [30].

In the future, accurate data from DBC’s and financial information could automatically prefill quality registry items. The DICA quality registry items are now entered manually, which is time-consuming and prone to registration errors. Reusing these data sources will lower the registration burden, reduce missing data, and validate data. These data can be used to complete registries and reduce hospital differences. Furthermore, RWD can also be used in health technology assessment decisions. This will be explored in the near future within European Union programs [31]. However, other data sources, such as pathology databases, must be linked to enrich the data. This additional data on histopathology and mutation status are essential as certain medications targeting specific mutations can influence outcomes. To improve shared decision-making, additional data sources, including patient-reported outcome measurements, must be linked to existing data sources.

### Conclusions

The DICA Medicines Program has shown that it is possible to gather and link RWD sources pertaining to medicines. In addition, these data became available with minimal registration burden and effort for hospitals. This method of providing RWD can be used in other population-based registries. The DICA Medicines Program provided participating centers with benchmark information and tools to evaluate the effectiveness of expensive medicines in real-world settings.

## Appendix

Multimedia Appendix 1 The medication page of the dashboard shows the active ingredients in a selected patient population and the percentage of patients treated with that active ingredient versus the benchmark. This page also shows the trend over the years. This overview can be adjusted with the filters to show the use of medicines in a specific population or year. Furthermore, it is also possible to receive an overview of the medicines with the number of courses instead of usage. The patients list contains the data of all patients that are selected for the shown results.

Multimedia Appendix 2 The signals page of the dashboard shows the insights of the dashboard in which a hospital deviates from the benchmark. There are also some specific signals to stimulate improving patient care.

Multimedia Appendix 3 There is a deep-dive function for every medicine in the dashboard that shows detailed information about the use of these medicines. The deep-dive also shows baseline patient and tumor characteristics of patients treated with this specific medicine, compared to the benchmark.

Multimedia Appendix 4 Details on the diagnostics are included per medicine in the dashboard for the hospital versus the results in the benchmark. These are related to the moment of medicine use (pre- or post treatment).

Multimedia Appendix 5 The costs page of the dashboard shows the total costs and the costs per patient for the hospital and the benchmark. This page can be adjusted for specific patient populations by using the filters.

## Abbreviations

CT: computerized tomography
DBC: diagnosis treatment combinations
DCRA: Dutch Colorectal Audit
DICA: Dutch Institute for Clinical Auditing
NBCA: National Breast Cancer Organization Breast Cancer Audit
NVZA: Dutch Association for Hospital Pharmacists
OS: overall survival
RCT: randomized controlled trial
RWD: real-world data
TTNT: time-to-next-treatment
VGZ: Institute of Public Healthcare
