# Supplementary figures and images for: Medication Use and Clinical Outcomes by the Dutch Institute for Clinical Auditing Medicines Program: Quantitative Analysis
Source: J Med Internet Res. 2022 Jun 23;24(6):e33446. doi: 10.2196/33446 (PMC9264125; doi:10.2196/33446)

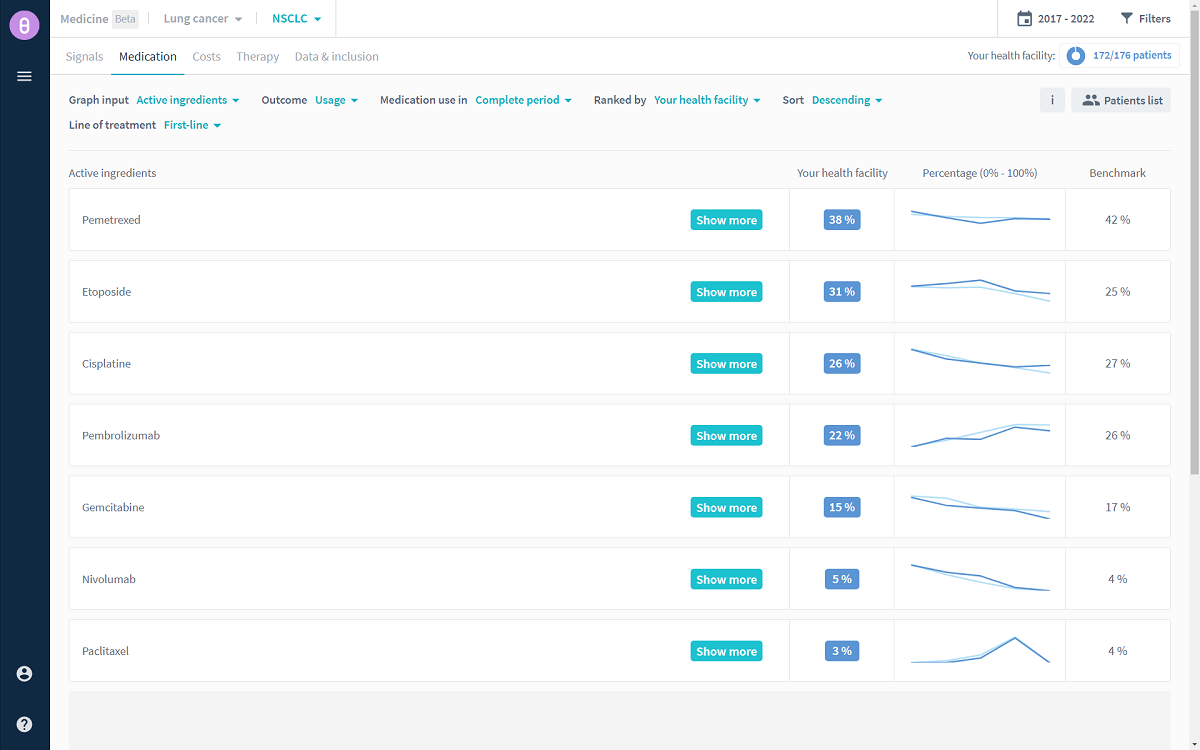

Supplement: Multimedia Appendix 1 [file jmir_v24i6e33446_app1.png]

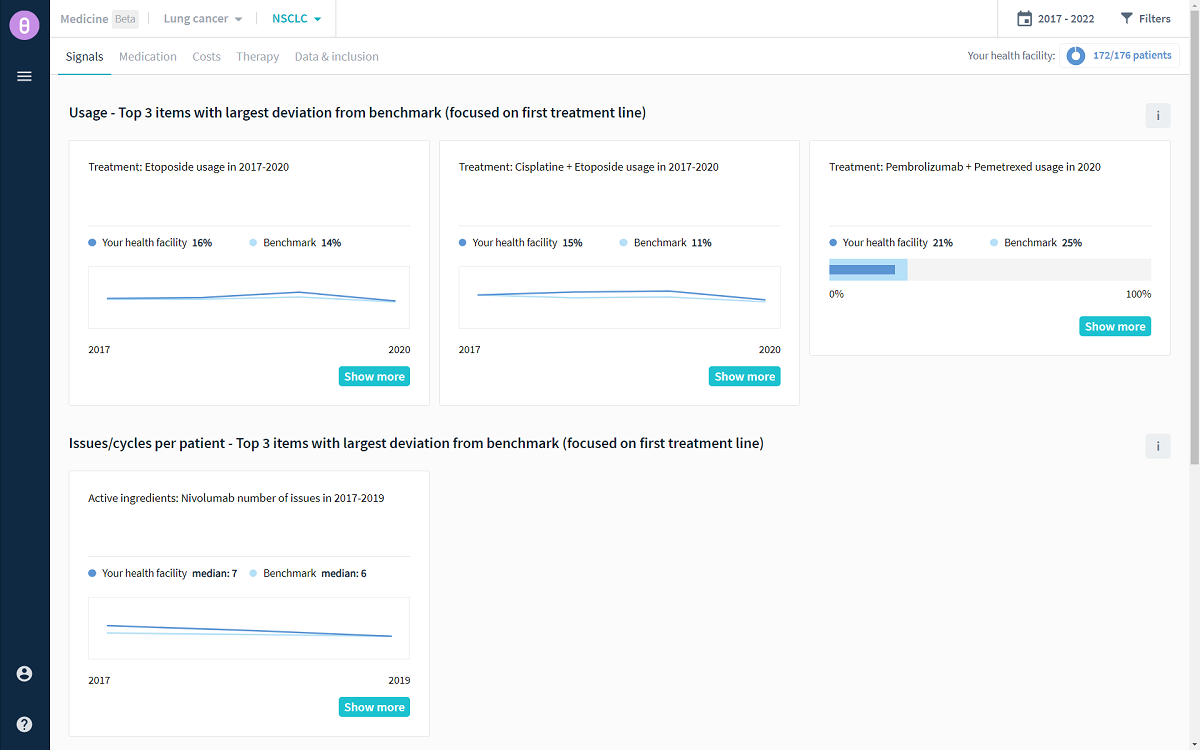

Supplement: Multimedia Appendix 2 [file jmir_v24i6e33446_app2.png]

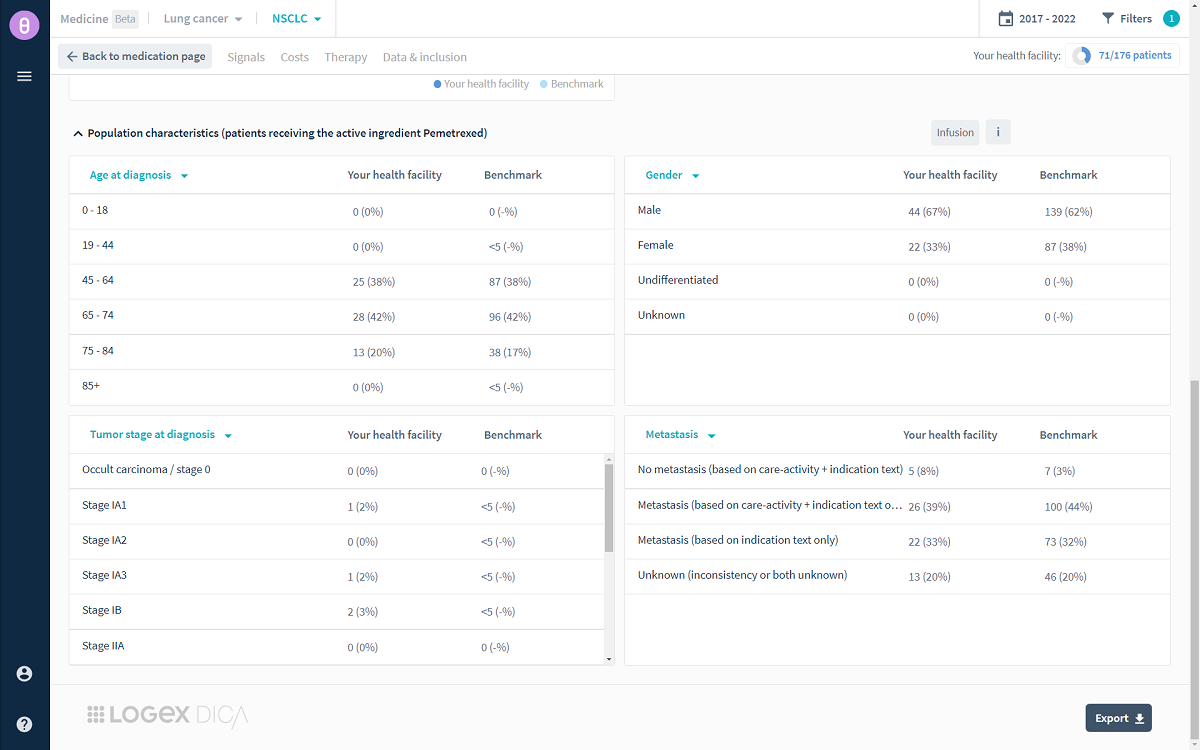

Supplement: Multimedia Appendix 3 [file jmir_v24i6e33446_app3.png]

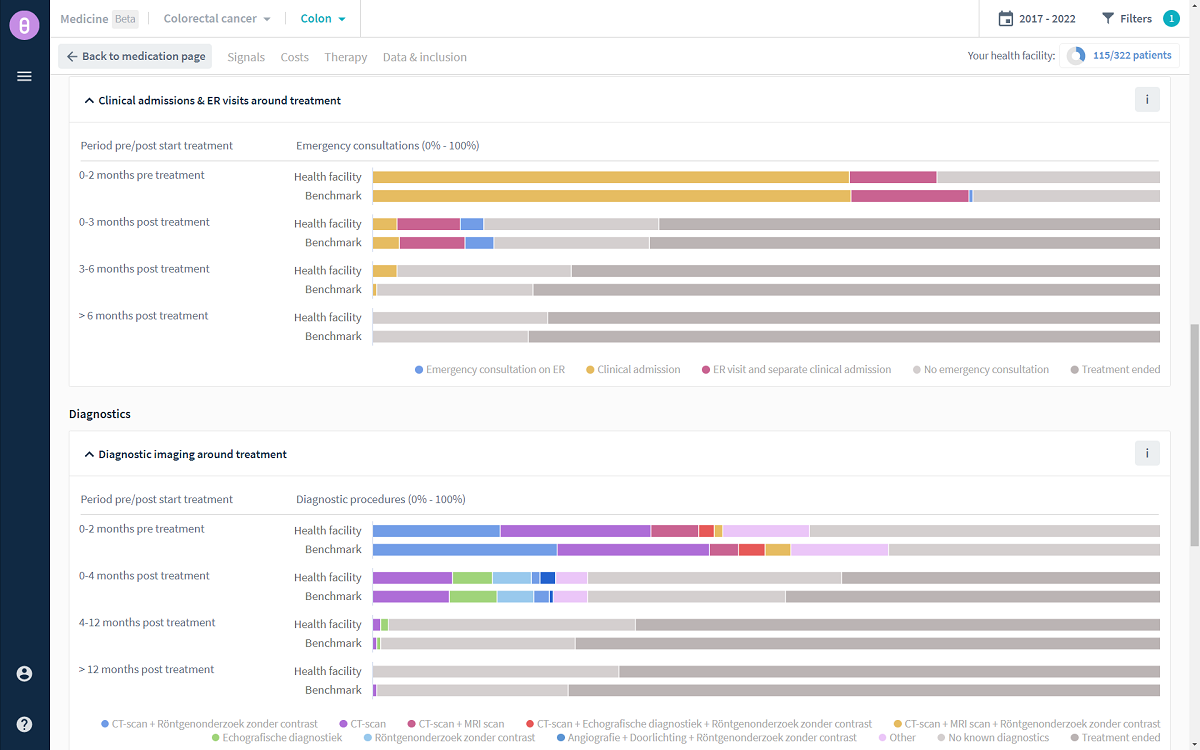

Supplement: Multimedia Appendix 4 [file jmir_v24i6e33446_app4.png]

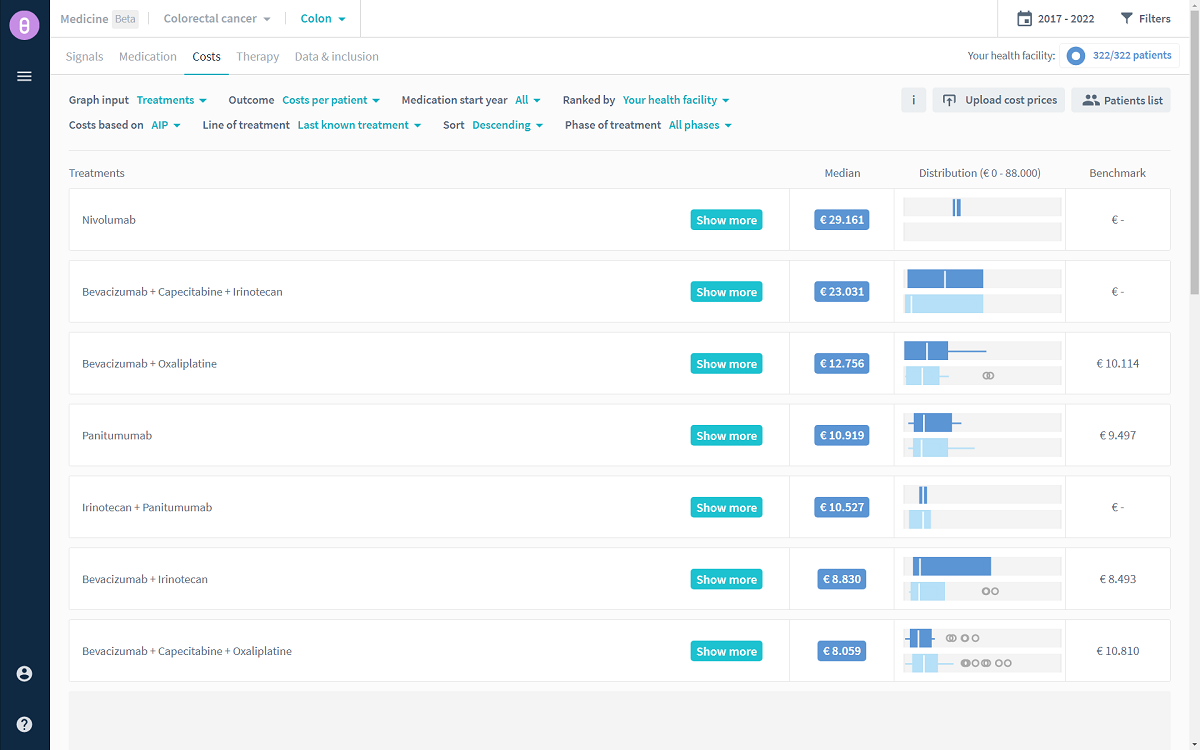

Supplement: Multimedia Appendix 5 [file jmir_v24i6e33446_app5.png]
